# A six-member SNP assay on the iPlex MassARRAY platform provides a rapid and affordable alternative for typing major African *Staphylococcus aureus* types

**DOI:** 10.1099/acmi.0.000018

**Published:** 2019-05-03

**Authors:** Justin Nyasinga, Cecilia Kyany’a, Raphael Okoth, Valerie Oundo, Daniel Matano, Simon Wacira, Willie Sang, Susan Musembi, Lillian Musila

**Affiliations:** 1 Kenyatta University, Nairobi, Kenya; 2 Technical University of Kenya, Nairobi, Kenya; 3 United States Army Medical Research Directorate – Africa, Nairobi, Kenya; 4 Kenya Medical Research Institute, Nairobi, Kenya

**Keywords:** *S. aureus*, typing, MassARRAY, *spa*, MLST, Kenya

## Abstract

**Purpose:**

Data on the clonal distribution of *Staphylococcus aureus* in Africa are scanty, partly due to the high costs and long turnaround times imposed by conventional genotyping methods such as *spa* and multilocus sequence typing (MLST), which means there is a need for alternative typing approaches. This study evaluated the discriminatory power, cost of and time required for genotyping Kenyan staphylococcal isolates using iPlex MassARRAY compared to conventional methods.

**Methodology:**

Fifty-four clinical *S. aureus* isolates from three counties were characterized using iPlex MassARRAY, *spa* and MLST typing methods. Ten single-nucleotide polymorphisms (SNPs) from the *S. aureus* MLST loci were assessed by MassARRAY.

**>Results:**

The MassARRAY assay identified 14 unique SNP genotypes, while s*pa* typing and MLST revealed 22 *spa* types and 21 sequence types (STs) that displayed unique regional distribution. *spa* type t355 (ST152) was the dominant type overall while t037/t2029 (ST 241) dominated among the methicillin-resistant *S. aureus* (MRSA) isolates. MassARRAY showed 83 % and 82 % accuracy against *spa* typing and MLST, respectively, in isolate classification. Moreover, MassARRAY identified all MRSA strains and a novel *spa* type. MassARRAY had a reduced turnaround time (<12 h) compared to *spa* typing (4 days) and MLST (20 days). The MassARRAY reagent and consumable costs per isolate were approximately $18 USD compared to *spa* typing ($30 USD) and MLST ($126 USD).

**Conclusion:**

This study demonstrated that iPlex MassARRAY can be adapted as a useful surveillance tool to provide a faster, more affordable and fairly accurate method for genotyping African *S. aureus* isolates to identify clinically significant genotypes, MRSA strains and emerging strain types.

## Introduction

Methicillin-resistant *Staphylococcus aureus* (MRSA) is a recognized global threat in the clinical management of infections caused by the pathogen [1, 2], which can cause a wide range of clinical syndromes [3]. Particular strains of MRSA tend to localize in certain geographical regions of the world, for example, USA1OO and USA3OO in North America [2] and EMRSA15 and EMRSA16 in the United Kingdom [4]. Strain typing can therefore be a useful tool for tracking high-risk *S. aureus* strains in a region. However, data on the clonal diversity and distribution of MRSA in Africa are scanty and warrant further studies [1, 5, 6]. For example, in Kenya, infections caused by *S. aureus *, including MRSA, are widespread [6–8], yet only two hospital studies have reported on the molecular epidemiology of the pathogen. In the first study, Omuse *et al*. reported the presence of major global MRSA clones such as MLST-CC5, 22, 7 and 30 among a heterogeneous collection of *S. aureus* isolates [6] and the predominance of members of MLST-CC5, such as ST1, ST5, ST8 and ST241. The second study reported that *spa* type t037 (ST239) was the dominant strain among clinical MRSA isolates [8].

High costs, long turnaround times for results, technical complexity and lack of technical expertise have impeded the adoption of conventional typing methods for surveillance studies, especially in resource-limited settings such as Africa [1, 5, 6]. In recent years, SNP-based genotyping has been proposed as a robust, efficient and low-cost approach to epidemiological characterization of *S. aureus* using methods such as iPlex MassARRAY [9, 10]. Despite its potential, iPlex MassARRAY has not been extensively evaluated against conventional methods such as *spa* typing and MLST, or against *S. aureus* strains from Africa whose epidemiology has been thought to be unique [5].

In this study, the results of a multiplexed SNP assay on the MassARRAY platform for a collection of Kenyan clinical *S. aureus* isolates are reported. In addition, comparisons between iPlex MassARRAY, *spa* and MLST with regard to discriminatory power, assay turnaround times, and reagent and consumable costs are presented.

## Methods

### Study population and isolates

This study analysed 54 archived clinical *S. aureus* isolates that had been recovered from samples collected sequentially between 2015 and 2016 from a recruited patient population of 221 persons. The isolates were obtained as part of an ongoing antimicrobial resistance surveillance project covering hospitals in three counties in Kenya where patients ≥2 months presenting with blood, urine, throat or skin and soft tissue infections are recruited. Standard microbiological techniques were used for the culture, isolation and identification of *S. aureus *. Isolate identities were further confirmed by conventional PCR for the *femA* gene [11]. Antimicrobial susceptibility testing was performed using both the Siemens MicroScan WalkAway 96 plus (Siemens Healthcare Diagnostics, Inc., New York, USA) and Kirby–Bauer disc diffusion methods. MRSA isolates were tested for the presence of the *mecA* and SCC*mec* types [12].

Of the 54 isolates, 16 were from Nairobi county, while Kisumu and Kericho counties were each represented by 19 isolates. Eighty-seven per cent (47/54) of the isolates were associated with community-acquired infections, defined as infections detected in the patient at presentation or within 48 h of hospitalization. Skin and soft tissue infections (SSTIs) contributed 94 % (51/54) of all isolates, while urinary tract infections accounted for 5.5 % (3/54). Eleven per cent (6/54) of the isolates were classified as MRSA. All MRSA isolates were *mecA*-positive and 4/6 had the SCC*mec* type IV genotype. All of the isolates were preserved as glycerol stocks at −80 °C.

### DNA extraction

All of the clinical isolates and four reference isolates [* Staphylococcus aureus* ATCC 43300, ATCC 25923, ATCC 25213 and CO-34 (NR-46191) (American Type Culture Collection and www.beiresources.org)] were sub-cultured by streaking a loopful of inoculum on Muller–Hinton agar plates (Sigma-Aldrich, St Louis, Missouri, USA) and incubating it for 18–24 h at 37 °C. The ZR/Fungal/Bacterial DNA MiniPrep kit (Zymo Research, Irvine, California, USA) was used for extraction following the manufacturer’s instructions, with two modifications: the processing time for the cell disruption step was set at 10 min and DNA was eluted with 60 µl of elution buffer. DNA was quantified using a Qubit DsDNA quantification kit (Thermo Fisher Scientific, Waltham, Massachusetts, USA). For the iPlex MassARRAY assays, DNA concentrations were normalized to 10 ng µl^−1^. DNA samples were stored at −20 °C.

### PCR amplification of the *spa* gene

Published primers were used to amplify the X region of the *spa* gene [13]. Each 25 µl PCR reaction mix included 10.5 µl of sterile nuclease-free water, 12.5 µl of Dream *Taq* mix (Thermo Fisher Scientific,Waltham, Massachusetts, USA) and 0.5 µl (10 pmol) of both the forward and reverse primers with 1 µl (approximately 50 ng) of template DNA. Cycling was performed in a GeneAmp 9700 PCR System (Applied Biosystems, Foster City, California, USA) with the following conditions: initial denaturation at 95 °C for 5 min, and 35 cycles of denaturation at 95 °C for 45 s, primer annealing at 60 °C for 30 s, extension at 72 °C for 90 s followed by a final extension at 72 °C for 10 min. Amplification products were resolved on a 1.5 % agarose in 1× Tris Acetate EDTA buffer (Sigma-Aldrich, St Louis, Missouri, USA) for 60 min at 95V and visualized with EZ Vision DNA stain (Amresco, Inc., Cleveland, Ohio, USA) using a UV transilluminator (UVP LLC, Upland, California, USA).

### PCR amplification of MLST loci

Published primers were used to amplify the MLST loci [14]. Each 25 µl reaction comprised 10.5 µl of sterile nuclease-free water, 12.5 µl of Dream *Taq* mix (Thermo Fisher Scientific, Waltham, Massachusetts, USA) and 0.5 µl (10 pmol) of both forward and reverse primers with 1 µl (approximately 50 ng) of template DNA. Thermocycler conditions were set as: initial denaturation at 95 °C for 3 min and 35 cycles of strand denaturation at 95 °C for 30 s, primer annealing at 55 °C for 30 s and extension at 72 °C for 60 s with a final extension at 72 °C for 10 min. The amplicons were resolved and visualized as described above for the *spa* gene.

### Sanger sequencing of *spa* and MLST amplicons

*spa* and MLST amplicons were purified using the DNA Clean and Concentrator kit (Zymo Research, Irvine, California, USA) following the manufacturer’s instructions with a final elution volume of 30 µl. Both forward and reverse strands were sequenced. Each reaction contained 4 µl of sterile distilled water, 2 µl of 5X Big Dye buffer (Applied Biosystems, Foster City, California, USA), 1 µl (4 µM) of the PCR primer, 1 µl of Big Dye terminator mix (Applied Biosystems, Foster City, California, USA) and 4 µl of amplicon DNA. Cycle sequencing was performed on an ABI 9700 thermocycler with cycling conditions set as: 94 °C for 5 min followed by 30 cycles of 94 °C for 15 s, 55 °C for 30 s and 68 °C for 2.5 min with a final extension of 68 °C for 3 min. Sequencing fragments were purified using Sephadex G50 resin (Sigma-Aldrich, St Louis, Missouri, USA) before loading on the Applied Biosystems 3500 Genetic Analyzer.

### Iplex MassARRAY assays for MLST SNPs

The 10 MLST SNPs used in this study have been reported previously [9]. Nine of the 10 MLST SNPs assayed were bi-allelic, while 1 (arcC210) was tri-allelic. The amplification and extension primers were designed using Agena Bioscience Assay Design Suite version 2.0 (Agena Bioscience, Hamburg, Germany). The sequences used to design the primers for primary amplification and extension PCR reactions were downloaded from the staphylococcal MLST database, http://www.mlst.net (20th June 2016) [14]. The primer sequences are shown in Table 1. All primary PCR primers had a 10-nucleotide tag (ACGTTGGATG) on the 5′ end to exclude them from the 4500–9000 Da mass range of matrix-assisted laser desorption/ionization time-of-flight mass spectrometry (MALDI-TOF MS) detection. The primary multiplex PCR reactions were run on a GeneAmp 9700 PCR machine (Applied Biosystems, Foster City, California, USA). Each reaction mix contained 1× PCR Buffer, 0.1 µM of forward and reverse primers, 4 mM of MgCl_2_, 500 µM of dNTP mix, 0.5 units of *Taq* polymerase and 10 ng of DNA template. The PCR conditions were set as follows: initial denaturation at 95 °C for 2 min, 25 cycles of 95 °C for 30 s, 56 °C for 30 s and 72 °C for 60 s, followed by a final extension at 72 °C for 5 min.

**Table 1.** Primary amplification primer sequences for various SNPs and their expected amplicon sizes

SNP ID*	Forward primer sequence (5′−3′)**	Reverse primer sequence (5′−3′)**	Amplicon length (bp)
arcC162	GTTGGGTTATCAAATCGTGG	ATAGTGATAGAACTGTAGGC	96
arcC210	GAGTCTGGCTGTTCTTTTTG	GATAAAGATGATCCACGATTC	118
aroE132	GGCTTTAATATCACAATTCC	GCACCTGCATTAATCGCTTG	103
aroE252	TCTGGATAAACGCTGTGCAA	AAGATGGCAAGTGGATAGGG	93
gmk129	AACTAGGGATGCGTTTGAAG	GGTGTACCATAATAGTTGCC	104
pta294	GATTAGTAAGTGGTGCAGCG	CCAGATGTTTTTGTAACGCC	114
tpi36	ATTCCACGAAACAGATGAAG	ACGCTCTTCGTCTGTTTCAC	120
tpi241	ATGAGCCAATCTGGGCAATC	GTTTGACGTACAAATGCACAC	102
tpi243	TGAACCAATCTGGGCAATCG	GTTTGACGTACAAATGCACAC	101
yqil333	CAACCATTGATTGATGTCCC	GGCGGTATGGAGAATATGTC	105

* arcC, carbamate kinase; aroE, shikimate dehydrogenase; gmk, guanylate kinase; pta, phosphate acetyl transferase; tpi, triosephosphate isomerase; yqil, acetyl co-enzyme A acetyl transferase.

** All amplification primers had a 5′ 10-mer tag.

Shrimp alkaline phosphatase (SAP) enzyme was used to dephosphorylate unused dNTPs from the primary PCR reaction before the second allele-specific primer extension (ASPE) PCR. For ASPE reactions, 2 µl of the reconstituted extension primer cocktail was added to each reaction well and the following conditions were set: initial denaturation at 94 °C for 30 s, 40 cycles of one step at 94 °C for 5 s with 5 sub-cycles of 52 °C for 5 s and 80 °C for 5 s, and a final extension at 72 °C for 3 min. The sequences and masses for unextended primers (UEPs) and extension products for each SNP and SNP allele are shown in Table 2. Extension products were conditioned using a resin, after which 10 nl was dispensed into a 96-well spectroCHIP using the MassARRAY Nanodispenser RS1000 (Agena Bioscience, Hamburg, Germany).

**Table 2.** Allele-specific extension primer sequences and expected masses for various SNP alleles

SNP ID	Alleles	Unextended primer (UEP) sequence (5′−3′)*	UEP mass (Da)	Call 1	Mass 1 (Da)	Call 2	Mass 2 (Da)
arcC162	T/A	CTGTAGGCACAATCGT	4881.2	A	5152.4	T	5208.3
arcC210	C/T/A	cTCCACGATTCAATAACCCAAC	6592.3	C	6839.5	T	6919.4
aroE132	A/G	agGATCTAAATACGGTATGATACG	7424.9	G	7672	A	7752
aroE252	T/A	ACAGATGGTATTGGTTATGT	6202	A	6473.3	T	6529.1
gmk129	C/T	CAGCATATTCTATAAATTGGTCATCTTT	8527.6	T	8798.8	C	8814.8
pta294	A/C	TGCTGGACGTACAGTATC	5514.6	C	5801.8	A	5841.7
tpi36	C/T	agCATTCCATGTTTGAAAATAGC	7046.6	T	7317.8	C	7333.8
tpi241	G/A	AATGCACACATTTCATTTG	5761.8	G	6009	A	6088.9
tpi243	A/G	CAAATGCACACATTTCATT	5730.8	G	5978	A	6057.9
yqil333	C/T	gctgGTCAACAACAGTCGCTT	6406.2	C	6653.4	T	6733.3

*The bases in lowercase were incorporated into the oligonucleotide sequences during primer design to prevent the extension products for various SNP alleles from being too close to each other in the mass spectrum.

### Costing and time comparisons

The reagent and consumable costs per isolate for *spa* typing and MLST were estimated based on local pricing for the items in each step in the processes: DNA extraction, primer sequences, PCR amplification, gel electrophoresis, PCR clean-up, cycle sequencing, fragment purification and fragment analysis. For the iPlex MassARRAY, additional costs for primer sequences, primary and allele-specific PCR amplifications, SAP treatment, sample conditioning and liquid transfer to spectrochips were estimated. Assay time comparisons for the three methods were also made. Technician time was not computed in the cost analysis, but it can be inferred from the assay times.

### Data analysis

Raw sequence chromatograms were examined using Chromas version 2.6.2 (Technelysium Pty Ltd, Brisbane, Australia) before consensus sequences were created using BioEdit Sequence Alignment Editor version 7.2.5 [15]. *spa* types were assigned using the online s*pa* type finder/identifier software, http://spatyper.fortinbras.us/ (Fortinbras Research). s*pa* types were further confirmed using the Ridom *spa* Server, https://www.spaserver.ridom.de/ (Ridom GmbH, Würzburg, Germany) [16]. For MLST loci, consensus sequences were aligned using the online alignment tool, MAFFT version 7 https://mafft.cbrc.jp/alignment/server/. Sequence types were assigned on the MLST database, www.mlst.net [14], using the ‘Exact or Nearest Match’ option. The SpectroAcquire program was used for data acquisition on the MassARRAY Compact Analyzer (Agena Bioscience, Hamburg, Germany) and detection parameters were set at 10 laser shots per raster position with a threshold of 5 good spectra per sample pad. In estimating the accuracy of iPex MassARRAY, all isolates were listed with their SNP genotypes and corresponding *spa* and MLST sequence types (STs). *Spa* and MLST classifications were used as the reference methods. The accuracy of iPlex MassARRAY was determined as the proportion of isolates whose SNP genotypes corresponded to particular *spa/*MLST types without ambiguity.

### Data availability

Raw chromatograms for *spa* and MLST typing as well as data output from the MALDI-TOF MS Compact Analyzer will be made available upon request.

## Results

### Distribution of *spa* and MLST types

*spa* and MLST typing yielded congruent results with 22 *spa* types and 21 STs respectively. The isolates displayed considerable heterogeneity with 16/22 *spa* types and 13/21 sequence types being represented by only one isolate. The three counties showed unique genetic fingerprints with minimal overlaps in isolate composition. Only t355 (ST152) was observed across the three counties with 18 *spa* types and 18 STs associating with specific counties. Kericho had 12 *spa* types (ten STs) while Kisumu had 10 *spa* types (eight STs) and Nairobi had five *spa* types (seven STs). Two novel *spa* types t17841 and t17826 were reported and were both associated with MSSA isolates. Despite the heterogeneity, t355 (ST152), t064 (ST8), t005 (ST22), t2029/t037 (ST241) were the dominant types. The six MRSA isolates were represented by types: [t2029 (ST241) *n*=1, t037 (ST241) *n*=3, t355 (ST152) *n*=1 and t007 (ST39) *n*=1].

### Typability and variability of the MLST SNPs

Table 3 summarizes the characteristics of the 10 SNPs analysed. Nine of the 10 SNPs interrogated were highly typeable, with SNP call rates (number of isolates in which a particular SNP was identified as a proportion of the total number of isolates tested) that ranged from 81–98.3 % (average 89%). One SNP, pta294, was only identified in one isolate and therefore could not be used to discriminate between isolates and was subsequently excluded from the analyses. Eight of the remaining SNPs were highly variable, with allele frequencies (number of isolates positive for a given SNP allele as a proportion of all isolates in which that SNP was identified) ranging from 53 % (arcC162) to 84.6 % (yqil333).

**Table 3.** Summary of the 10 MLST SNPs analysed by iPlex MassARRAY

SNP	Polymorphism	SNP call rate^*a*^	Allele frequency^*b*^
arcC162	T/A	98.3% (57/58)	**T** 53% (30/57)
arcC210	C/T/A	86% (50/58)	**T** 60% (30/50)
aroE132	A/G	91.3% (53/58)	**A** 73.6% (39/53)
aroE252	T/A	86% (50/58)	**T** 64% (32/50)
gmk129	C/T	84.5% (49/58)	**C** 67.3% (33/49)
tpi36	C/T	81% (47/58)	**C** 63.8% (30/47)
tpi241	G/A	86% (50/58)	**G** 98% (49/50)
tpi243	A/G	94.8% (55/58)	**G** 60% (33/55)
yqil333	C/T	89.7% (52/58)	**T** 84.6% (44/52)
pta294	A/C	–*	–*

*pta294 was only identified in one isolate and was excluded when generating SNP genotypes.**** ****

*a,* SNP call rate: The number of isolates in which a given SNP was identified as a proportion of all isolates tested for that SNP.

*b*, Allele frequency: The number of isolates in which a given SNP allele was identified as a proportion of all isolates in which the SNP was identified.

### Iplex MassARRAY SNP genotypes

The combination of nine SNPs for each isolate was used to generate SNP genotypes. To identify the smallest number of SNPs with the highest resolution, different SNP combinations were created by sequentially and progressively reducing the number of SNPs while comparing the resultant classifications with those of the full nine-member SNPs. First, individual SNPs were excluded to create eight-member SNP classifications. Four SNPs (arcC162, arcC210, gmk129 and yqil333) proved to be individually discriminative, while the remaining five (aroE132, aroE252, tpi36, tpi241 and tpi243) contributed to the resolution by acting in combination (Table S1, available in the online version of this article). Next, different SNP pairs from the five SNPs that had combinatorial resolution were excluded to create seven-member SNP classifications. Six different combinations of seven SNPs had a similar resolution to that of the nine-member classification. Lastly, different sets of three SNPs from the five SNPs with combinatorial resolution were excluded to create six-member SNP groupings. In the end, a minimum of six SNPs was required to achieve the resolution of the nine-member SNP profiles. Two unique six-member SNP combinations were identified (arcC162, arcC210, aroE132, gmk129, tpi243 and yqil333 and arcC162, arcC210, gmk129, tpi36, tpi243 and yqil333). In this paper, classifications based on the former combination are presented.

**Fig. 1.**
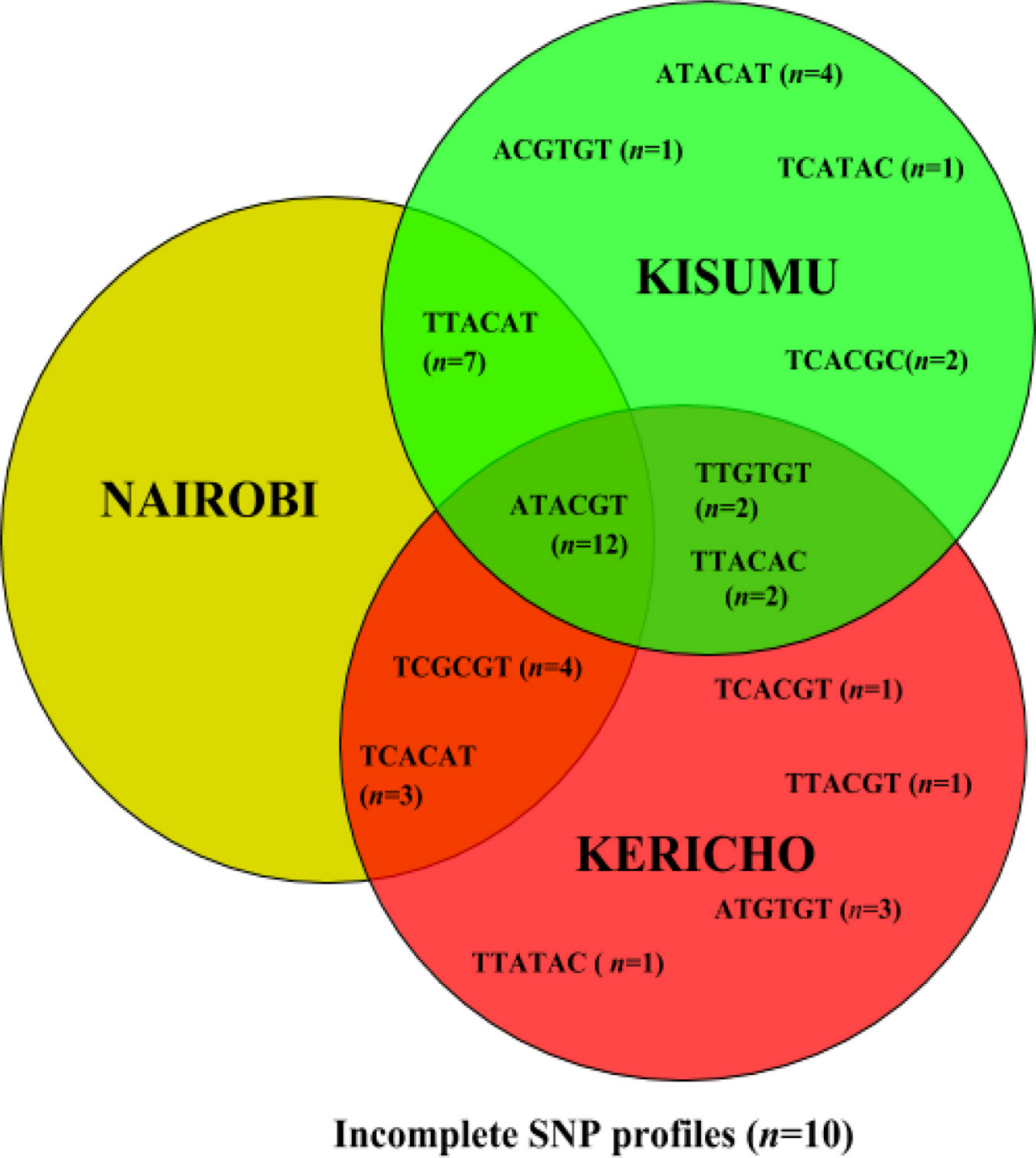
Distribution of *S. aureus* isolate types in the three counties by MassARRAY SNP genotyping. The six-member SNP genotypes are in the order of arcC162, arcC 210, aroE 132, gmk 129, tpi 243 and yqil 333. Isolates in which at least one of the seven SNPs used for genotype assignment was not identified were considered incomplete.

The assay grouped 44/54 isolates into 14 different SNP genotypes. Fig. 1 shows the regional distribution of various SNP genotypes. In Kericho, nine SNP genotypes were observed among the isolates, while the Kisumu and Nairobi isolates showed eight and four SNP genotypes, respectively. The assay successfully typed 11/16, 16/19 and 17/19 isolates from Nairobi, Kericho and Kisumu, respectively, while 10 isolates did not have complete SNP profiles. The classifications achieved by iPlex MassARRAY reflected the heterogeneity and regional distribution patterns revealed by *spa* typing and MLST.

The 14 SNP genotypes were compared with corresponding *spa* and MLST STs. The iPlex MassARRAY showed 83 % (34/41) accuracy against *spa* typing and 82 % (32/39) against MLST. The SNP genotype TTACAC corresponded to a novel *spa* type t17841. iPlex MassARRAY identified all MRSA isolates in this collection. One MRSA was of the ATACGT genotype, which corresponded to t355. t037, which was the *spa* type of three MRSAs, and t2029 (one MRSA) were represented by the ATACAT genotype. ACGTGT corresponded to t007 (one MRSA).

**Fig. 2.**
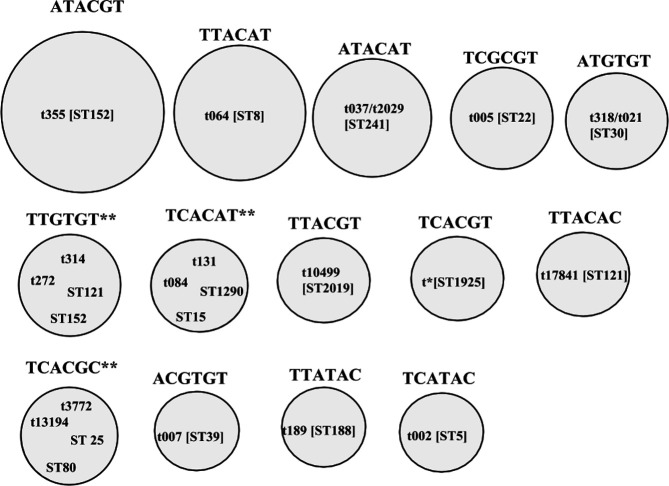
A comparison of the classifications between iPlex MassARRAY, *spa* typing and MLST. The six-member SNP genotypes indicated above each circle are in the order of arcC162, arcC 210, aroE 132, gmk 129, tpi 243 and yqil 333. The *spa* types are indicated with the prefix t and the MLST types indicated with the prefix ST. The different sizes of the circles represent the frequency of occurrence of various strain types. t*, an unassigned novel *spa* repeat sequence. **, SNP profiles in which discrepant classifications were observed.

Three discrepancies in the iPlex MassARRAY assay were observed: SNP genotype TCACGC could not distinguish between t3772 and t13194 (ST 25 and ST80); SNP genotype TTGTGT could not distinguish between t314 and t272 (ST121 and 152); and TCACAT could not distinguish between t084 and t131 (ST 15 and ST1290). Discrepancies involving *spa* types t318/t021 (ST30) and t037/t2029 (ST241) were considered to be minor, as the isolates are closely related and indistinguishable by MLST (Fig. 2).

### Turnaround time and reagent and consumable costs per isolate

The iPlex MassARRAY assay took approximately 1 day to complete compared to *spa* typing and MLST, which took approximately 4 and 20 days, respectively (Table 4) to complete. The reagent and consumable costs for analysing an isolate using MassARRAY were approximately $18 USD compared to $30 USD/isolate for *spa* typing and $126 USD/isolate for MLST for the collection of isolates analysed.

**Table 4.** Assay time comparisons for *spa*, MLST and MassARRAY typing methods

	*spa* typing		MLST	
Step	Time	Period	Time	Period
Amplification PCR	3 h	1 day	21 h	2 days
Gel electrophoresis	2 h		7 h	1 day
PCR amplicon clean-up	2 h		14 h	2 days
Sequencing PCR	3 h	1 day	21 h	2 days
Presequencing purification	3 h		21 h	2 days
Sequence fragment analysis	8 h	1 day	56 h	7 days
Data analysis	7 h	1 day	30 h	4 days
Total time	4 days		20 days

**Table:** 

	iPlex MassARRAY			
Step	Time	Period		
Amplification PCR	2.5 h			
SAP treatment	0.75 h			
iPlex PCR	3 h			
Sample conditioning	1 h			
Data acquisition	1 h			
Data analysis	3 h			
Total time		1 day		

## Discussion

Six MLST SNPs identified 14 unique genotypes and reflected the heterogeneity and distribution depicted by *spa* typing/MLST of a collection of Kenyan isolates. The isolate types ST152, ST5, ST8, ST15, ST30 and ST241 identified by MassARRAY have been reported in other African countries, such as Cameroon, Madagascar, Morocco, Niger, Senegal [17] and Ghana [18], highlighting the potential of this assay to be applied in the larger African context.

MassARRAY demonstrated the clear advantages of reduced turnaround time and reagent and consumable costs per isolate based on the time and costs in the Kenyan context, which could vary in different settings. In one study, when the iPlex MassARRAY assay costs were compared to SYBR green real-time PCR, there was a 60 % reduction in reagent costs [9]. Trembizki *et al*. noted an approximately 30 % reduction in costs compared to performing a full MLST analysis [19]. The cost of a MassARRAY platform is higher than that for conventional PCR machines and genetic analysers and therefore prohibitive for a single programme or project to procure, but is best borne as a core institutional capability shared by different projects. The high initial costs for the MassARRAY system are justified by the short turnaround times, multiplexing, automation and throughput capabilities, which can support multiple large-scale studies concurrently.

As the MassARRAY technology is increasingly being adopted, studies utilizing its application in bacterial genotyping are being reported. In one study, a 14-member SNP assay on the MassARRAY platform was developed for genetic characterization of *Neisseria gonorrhea* in Australia [19] and subsequently applied in a large-scale AMR surveillance study [20]. Even though only 10 SNPs were reported in this study, the technique can be designed to detect more SNPs for increased resolution and even identify binary gene markers to answer clinically important questions about a pathogen, for example regarding virulence and resistance [9].

While the increasing availability of whole-genome sequencing platforms and the concomitant reduction in sequencing costs is recognized, this assay remains superior in terms of the cost and speed of obtaining specific information, such as strain type and presence or absence of resistance or virulence genes, while avoiding the rigours of handling and analysing whole-genome sequencing data. Additionally, the assay would be most relevant for long-term surveillance studies, where it would be impractical to perform *spa* typing*,* MLST or whole-genome sequencing on hundreds to thousands of isolates.

In some instances, the assay could not distinguish between two or more *spa*/MLST types, an observation noted by others [21, 22]. However, the affected *spa*/MLST types did not belong to major circulating strains, as they were each represented by one isolate, none of which was an MRSA strain. Previously, it has been suggested that increasing the number of SNPs can resolve minor *S. aureus* clones [22]. While the potential of a larger SNP set to improve resolution is recognized, a different set of SNPs in addition to the six identified here may be required.

There were instances, for example with the pta294 SNP, where particular SNPs could not be called. A possible explanation for this could be sequence variation in the target locus for the primer in the primary PCR reactions, an observation that has been noted elsewhere [9, 19]. The quality of DNA can potentially affect the success of the assay [19], however, the DNA used for these experiments was of high quality, as extraction was performed using a commercial kit and DNA concentrations were measured and normalized.

Even though the SNPs analysed had generally high identification rates, the combinatorial nature of the method, which requires all SNP results for genotype assignment, meant that 10 isolates could not be classified despite having most of the alleles. This could be solved by further optimization of assay conditions and rescreening of the missing loci to complete the panel for classification.

The lack of a database for MLST SNPs that can be used for matching particular SNP profiles to known STs or *spa* types [19] is a major limitation. This is partly due to the lack of rigorously evaluated MassARRAY SNP data against known *spa* /STs from an international collection of isolates. With increased validation, it should be possible to have an online MLST-style platform where SNPs can be submitted for inter-laboratory comparability of data.

Compelling epidemiological conclusions cannot be drawn from this study due to the modest collection of 54 isolates analysed. A larger collection of isolates from diverse regions and clinical syndromes would not only give a reliable reflection of the epidemiology of *S. aureus* in Kenya but also serve to validate the utility of iPlex MassARRAY for surveillance.

In conclusion, six SNPs derived from the MLST loci provided comparable discriminatory power for resolving a heterogeneous and regionally unique collection of Kenyan clinical *S. aureus* isolates, including MRSA strains. The iPlex MassARRAY demonstrated the advantages of reduced turnaround time and assay costs compared to two conventional typing methods. With increased validation, the assay could serve as a complement to existing typing methods in *S. aureus* surveillance studies to establish the epidemiology of *S. aureus* strains and monitor for the presence or emergence of high-risk strains in a timely fashion. 

## Supplementary Data

Supplementary material 1
