# Supplementary material for: A six-member SNP assay on the iPlex MassARRAY platform provides a rapid and affordable alternative for typing major African Staphylococcus aureus types
Source: Access Microbiol. 2019 May 3;1(3):e000018. doi: 10.1099/acmi.0.000018 (PMC7471782; doi:10.1099/acmi.0.000018)
Supplement: Supplementary material 1 [file acmi-1-018-s001.pdf]

Supplementary table 1: Different SNP combinations and their comparisons to the full 9-member SNP classification

arcC 162	arcC 210	aroE 132	aroE 252	gmk 129	tpi36	tpi 241	tpi 243	yqil 333	Remark
**8-member SNP classifications**									
X	*	*	*	*	*	*	*	*	No distinction between t064 and t037/t2029
*	X	*	*	*	*	*	*	*	No distinction between t064 and t084/t131; t189 and t002
*	*	X	*	*	*	*	*	*	Classification similar to full 9-member SNP profile
*	*	*	X	*	*	*	*	*	Classification similar to full 9-member SNP profile
*	*	*	*	X	*	*	*	*	No distinction between t189 and t17841
*	*	*	*	*	X	*	*	*	Classification similar to full 9-member SNP profile
*	*	*	*	*	*	X	*	*	Classification similar to full 9-member SNP profile
*	*	*	*	*	*	*	X	*	Classification similar to full 9-member SNP profile
*	*	*	*	*	*	*	*	X	No distinction between t064 and t17841
**7-member SNP classifications**									
*	*	*	*	*	X	*	X	*	No distinction between t084/t131 and a novel repeat sequence
*	*	*	X	*	*	*	X	*	No distinction between t064 and t10499
*	*	X	*	*	X	*	*	*	No distinction between t005 and a novel repeat sequence
*	*	X	*	*	*	*	X	*	No distinction between t005 and t084/t131
*	*	X	X	*	*	*	*	*	Classification similar to full 9-member SNP profile
*	*	X	*	*	*	X	*	*	Classification similar to full 9-member SNP profile
*	*	*	X	*	X	*	*	*	Classification similar to full 9-member SNP profile
*	*	*	X	*	*	X	*	*	Classification similar to full 9-member SNP profile
*	*	*	*	*	X	X	*	*	Classification similar to full 9-member SNP profile
*	*	*	*	*	*	X	X	*	Classification similar to full 9-member SNP profile
**6-member SNP classifications**									
*	*	*	*	*	X	X	X	*	No distinction between t084/t131 and a novel repeat sequence

*	*	*	X	*	*	X	X	*	No distinction between t064 and t10499
*	*	*	X	*	X	*	X	*	No distinction between t355 and t037
*	*	*	X	*	X	X	*	*	Classification similar to full 9-member SNP profile
*	*	X	*	*	*	X	X	*	No distinction between t005 and t084/t131
*	*	X	*	*	X	*	X	*	No distinction between t005 and t084/t131
*	*	X	*	*	X	X	*	*	No distinction between t005 and a novel repeat
*	*	X	X	*	*	*	X	*	No distinction between t005 and t084/t131
*	*	X	X	*	*	X	*	*	Classification similar to full 9-member SNP profile
*	*	X	X	*	X	*	*	*	No distinction between t005 and a novel repeat sequence

SNPs that were included in various SNP combinations are denoted by (*****), while those SNPs that were excluded are shown by (X). The SNPs were sequentially and progressively reduced to create eight, seven and six SNP profiles in order to find the smallest number of SNPs with the highest discrimination.
